# Long non-coding RNA LINC00240 promotes gastric cancer progression via modulating miR-338-5p/METTL3 axis

**DOI:** 10.1080/21655979.2021.1983276

**Published:** 2021-11-29

**Authors:** Guoping Wang, Zhongchen Zhang, Chenmei Xia

**Affiliations:** Department of Endoscopy Center, First People’s Hospital of Wenling, Wenling, Zhejiang, China

**Keywords:** LINC00240, miR-338-5p, METTL3, gastric cancer, cell function

## Abstract

Gastric cancer (GC) is a common cancer with high incidence. Understanding the epidemiology and physiopathology of GC is crucial for formulating novel therapeutic strategies. Recent studies have implicated long non-coding RNA LINC00240, miR-338-5p and methyltransferase-like 3 (METTL3) in the progression of GC. In this study, we investigated the functional role of LINC00240/miR-338-5p/METTL3 axis in regulating the aggressiveness of GC cells. We first demonstrated that LINC00240 was upregulated in GC tissues and GC cell lines. High expression of LINC00240 was associated with advanced TNM stage, a higher extent of distant metastasis and lymph nodes metastasis, and the poor overall and disease-free survival of the patients. In GC cell lines, the knockdown of LINC00240 inhibited GC cell proliferation and migration, but induced cell apoptosis. We further identified and validated the functional interaction between LINC00240 and miR-338-5p. miR-338-5p seemed to function as a downstream target negatively regulated by LINC00240, and miR-338-5p could target METTL3 at 3ʹ UTR to downregulate its expression. In GC tissues, the expression of miR-338-5p was negatively correlated with LINC00240, and the expression of miR-338-5p was negatively correlated with METTL3. Importantly, miR-338-5p inhibitor or METTL3 overexpression could rescue the inhibitory effect of LINC00240 knockdown on cell proliferation and migration, and inhibit the apoptosis induction in GC cells. Taken together, our data imply that the upregulation of LINC00240 in GC cells promotes the malignant phenotype by modulating miR-338-5p/METTL3 axis, which could serve as potential therapeutic targets for GC treatment.

## 1. Introduction

According to GLOBOCAN 2018 data, gastric cancer (GC) is the third leading cause of cancer-related deaths worldwide [1,2]. Although the decline of *Helicobacter pylori* infection rate has decreased the incidence of GC in the developed countries, the incidence and mortality are still high in developing world such as Asia, Eastern Europe and South America [1,2]. Therefore, understanding the mechanisms regulating the physiopathology of GC is crucial for formulating novel therapeutic strategies.

Long non-coding RNAs (lncRNAs) are the non-coding transcripts regulating gene expression at transcriptional and post-transcriptional levels, which are implicated in multiple physiological and pathological processes [3,4]. Particularly, accumulating evidence has highlighted the functional roles of lncRNAs in modulating tumorigenesis, metastasis and tumor suppression. Among the identified lncRNAs, HOX transcript antisense RNA (HOTAIR) and metastasis associated in lung adenocarcinoma transcript 1 (MALAT1) are the best characterized examples in cancer progression regulation [5–8]. Recently, Ku et al. reported that LINC00240 suppresses the invasion of lung cancer cells by modulating miR-7-5p/epidermal growth factor receptor (EGFR) axis in non-small cell lung cancer [9]. Moreover, Li et al. observed the upregulation of LINC00240 expression in GC tissues and cells [10]. The study further showed that LINC00240 promotes cell proliferation, migration and epithelial–mesenchymal transition (EMT) via regulating miR-124-3p/DNA methyltransferase 3 beta (DNMT3B) axis. However, the functional involvement of other downstream targets of LINC00240 in GC progression remain to be elucidated.

As a type of small non-coding RNAs, microRNAs (miRNAs) regulate gene expression via directly binding with mRNAs. miR-338-5p has been recognized as a tumor suppressor which suppresses cell proliferation, migration and induces apoptosis in different cancers, such as esophageal squamous cancer, glioblastoma and gastric cancer [11–15]. Particularly, several studies have demonstrated the tumor suppressor function of miR-338-5p in GC cells [14–16]. However, the functional relationship between LINC00240 and miR-338-5p in GC has not been studied yet.

Methyltransferase-like 3 (METTL3) is an m6A methyltransferase which may function as an oncogenic factor or a tumor suppressor in various types of cancer [17]. In GC, METTL3 has been recognized as a potential prognostic marker, promoting the malignant phenotype of GC cells and metastasis via EMT and tumor angiogenesis [18–21]. Wu et al. recently reported that miR-338-5p inhibits proliferation and migration of lung cancer cells by modulating METTL3/c-Myc axis [22]. However, the other regulators of METTL3 in GC have not been fully revealed.

In this study, we investigated the role of LINC00240 and its molecular mechanisms on GC cell growth, proliferation and invasion. We focused on the functional role of LINC00240/miR-338-5p/METTL3 axis in GC based on bioinformatic target prediction and previous literatures. Our results showed that LINC00240 plays a critical role in promoting GC cell proliferation and invasion via modulating miR-338-5p/METTL3 axis, which enriches the regulatory network of LINC00240 and provides potential therapeutic targets for GC treatment.

## 2. Materials and methods

### 2.1. Tissue samples and cell culture

GC tissues and adjacent normal tissues (n = 60) were collected from the GC patients by surgery at First people’s hospital of Wenling, Zhejiang province, China. The patients enrolled in this study had no history of chemotherapy and radiotherapy. This study was approved by the Ethics Committee of First people’s hospital of Wenling (WL/2017-5998). All patients signed the informed consent.

Four GC cell lines (SGC-7901, BGC-823, AGS and MKN45) and human normal gastric epithelial cell line (GES 1) were used in our study (ATCC, Manassas, VA, USA). SGC-7901, BGC-823 and MKN45 cells were cultured in RPMI-1640 medium (ATCC) with 10% FBS (ATCC) and antibiotics (100 U/ml penicillin and 100 μg/ml streptomycin, Corning, NY, USA), AGS cells were cultured in ATCC-formulated F-12 K medium (ATCC) with 10% FBS and the above antibiotics. All the cells were cultured in a humidified incubator with 5% CO_2_ at 37°C.

### 2.2. Cell transfection

GC cells were transfected with small interfering RNAs (siRNAs): si-LINC00240^1#^, si-LINC00240^2#^, or si-NC. To express METTL3, the cDNA sequences of METTL3 were cloned into pcDNA3.1 expression vector (Invitrogen). miR-338-5p inhibitor, miR-338-5p mimic and negative controls were purchased from Creative Biogene (NY, USA). Lipofectamine 3000 (Invitrogen) were used for small RNAs and plasmids transfection into GC cells according to the manufacturer’s instructions. Cells were used for functional experiments 48 hours post-transfection. The sequences of si-RNAs were: si-LINC002401#, UGUUCUUCCUGUAUUUCUGAG; si-LINC002402#, ACAUUAUUGAUGUUCUUCCUG.

### 2.3. Quantitative real-time PCR (qRT-PCR)

The total RNA was isolated using HighPrep™ RNA Kit according to the manufacture’s protocol (MagBio Genomics, USA). cDNA was synthesized using SuperScript IV One-Step RT-PCR System (Invitrogen). qRT-PCR was performed with QuantiTect Probe PCR Kits (QIANGEN, China) on a LightCycler 480 system (Roche, Switzerland). 2^−ΔΔCt^ method was used to quantify the relative gene expression level, with β-actin as the reference gene. The primers sequences used in the study were listed as below: METTL3-F: 5ʹ-CAAGCTGCACTTCAGACGAA-3ʹ; METTL3-R: 5ʹ-GCTTGGCGTGTGGTCTTT-3ʹ; miR-338-F: 5ʹ-CTCAACTGGTGTCGTGGAGTCGGCAATTCAG-3ʹ; miR-338-R: 5ʹ-TTGAGTTGTTATA-3ʹ; LINC00240-F: 5ʹ-GACTGCGATGGTTTGCAGAG-3ʹ; LINC00240-R: 5ʹ-GGAGTAGTTGAGGGTTGGCA-3ʹ; control gene: β-actin-F: 5ʹ-GTGGGCCGCTCTAGGCACCAA-3ʹ; β-actin-R: 5ʹ-CTCTTTGATGTCACGCACGATTTC-3ʹ.

### 2.4. Protein quantification by Western Blot analysis

The cells were first lysed and dissolved in RIPA buffer (Sigma-Aldrich) on ice. After centrifuging, the concertation of the total protein lysate in the supernatant was measured using BCA Protein Assay (Pierce, IL, USA). 50 µg of total protein from each sample was used for SDS-PAGE electrophoresis. Separated protein in SDS-PAGE gel was transferred onto PVDF membrane (Sigma-Aldrich). After blocking with 5% skimmed milk for 1 hour, the membrane was incubated with primary antibody at 4°C overnight (Anti-beta Actin antibody (ab8227, Abcam) used at 1:1000 dilution, Anti-METTL3 antibody [EPR18810] (ab195352, Abcam) used at 1:1000 dilution). The membrane was washed 3 times with TBST buffer for 5 minutes each. After wash, the membrane was further incubated with HRP-linked secondary antibody (1:3000; Cell signaling #7074, MA, USA) at room temperature for 1 hour. After washes with TBST buffer, the protein bands were visualized using ECL detection ECL detection kit according to the manufacture’s instruction (Pierce, IL, USA), and photographed on a gel imager system (Bio-Rad, Hercules, CA, United States). The densitometry analysis was performed with Image J software.

### 2.5. Cell viability, migration and invasion assays

For measuring cell proliferation, 48 hours after transfection, cells were seeded in to a 96-well plate at a density of 1500 cell/well and cultured in a humidified cell culture incubator for 0, 24, 48, 72 and 96 hours, respectively. Subsequently, 10 μL CCK8 reaction solution (ApexBio, TX, USA) was added to the cell culture at indicated time point and incubated for 1 hour in a humidified cell culture incubator. The light absorption value (OD value) was recorded at 450 nm wavelength on a Synergy H1 microplate reader (Winooski, Vermont, USA).

For cell migration and invasion assay, the transwell upper chamber (Corning, NY, USA, #3401) without Matrigel (BD Biosciences, MA, USA, # 356234) was used for migration assay, while transwell upper chamber coated with Matrigel was used for invasion assay. 5 × 10^5^ Cells were inoculated into the upper chamber in serum-free medium and 10% serum-containing medium was added to the lower chamber. After 18 hours, culture medium was discarded and the cells were fixed with 4% paraformaldehyde for 10 mins and stained with 0.5% crystal violet (Sigma, Germany, #109218) for 20 mins. Cells were photographed under Leica AM6000 microscope.

### 2.6. Flow cytometry analysis of apoptosis

The detection of cell apoptosis was performed using Annexin V-FITC Apoptosis Detection Kit (Sigma-Aldrich) according to the manufacturer’s instructions. In brief, 10 μL Annexin V-FITC and 5 μL PI were added to the 1000 μL cell resuspension with 5 million cells and incubated for 30 mins in the dark. Stained cells were centrifuged and washed twice with 1 PBS and resuspended in 400 μL PBS. The percentage of apoptotic cells was detected by BD FACS CantoTM II Flow Cytometer (BD Biosciences).

### 2.7. Dual luciferase reporter assay

The sequence containing the wild type binding site of miR-338-5p and LINC00240 and the sequence with mutated binding site were cloned into the PmirGLO vector expressing firefly luciferase (Promega, E1330). The reporter plasmid and Renilla luciferase (hRlucneo) control plasmid were co-transfected into cells with either miR-338-5p mimic or miR-NC in a 12-well plate (1 × 10^5 cells/well) using Lipofectamine 3000 reagent according to the manufacturer’s instructions (Invitrogen, L3000001). Similarly, the reporter vectors containing the wild type binding site of miR-338-5p and METTL3 and the mutated sequence were constructed and co-transfected with Renilla luciferase (hRlucneo) control plasmid in the presence of miR-338-5p mimic or miR-NC. 48 h post transfection, the relative luciferase activities were recorded using the Dual-Luciferase Reporter Assay Kit (Promega, E1910) on a luminescence microplate reader (Infinite 200 PRO; Tecan).

### 2.8. Statistical analysis and bioinformatics analysis

Statistical analysis was conducted using GraphPad Prism 8.0. All experiments were performed three times. One-way analysis of variance (ANOVA) was used to compare the difference among multiple groups. Two tailed Student’s *t*-test was used to compare the difference between two groups. Spearman correlation analysis was used to evaluate the correlation between LINC00240 and miR-338-5p expression, as well as the correlation between miR-338-5p and METTL3 expression. The results were presented as mean ± standard deviation. *p* < 0.05 was considered as statistically significant.

For TCGA data analysis, the online tool UALCAN (http://ualcan.path.uab.edu) was used to explore the expression of target genes. To identify the potential-binding partners of LINC00240, we first retrieved the sequence of LINC00240 in NCBI database (https://www.ncbi.nlm.nih.gov/gene). The sequence of LINC00240 was subject to miRNA target prediction using LncBase v.2 Prediction Module of DIANA Tools (http://carolina.imis.athena-innovation.gr/diana_tools/web/). To identify the target of miR-338-5p, TargetScan database (http://www.targetscan.org/vert_72/) was used to predict the mRNA targets containing potential-binding sites for miR-338-5p.

## 3. Results

In this study, we explored the functional role of LINC00240 in regulating the malignant phenotype of GC cells. We found that LINC00240 upregulation in GC tumors was associated with poor overall and disease-free survival of the patients. Loss-of-function experiment showed that knocking down LINC00240 inhibited GC cell proliferation and migration, but induced cell apoptosis. Through online bioinformatic tools, a binding site between miR-338-5p and LINC00240, as well as a binding site between miR-338-5p and METTL3 were identified. Their functional interaction was confirmed by dual luciferase reporter assay. In GC tissues, the expression of miR-338-5p was negatively correlated with LINC00240, and the expression of miR-338-5p was negatively correlated with METTL3. We further demonstrated that miR-338-5p inhibitor or METTL3 overexpression could rescue the inhibitory effect of LINC00240 knockdown on cell proliferation and migration, and inhibit the apoptosis induction in GC cells.

### 3.1. LINC00240 is upregulated in GC tissues and cell lines

**Figure 1.**
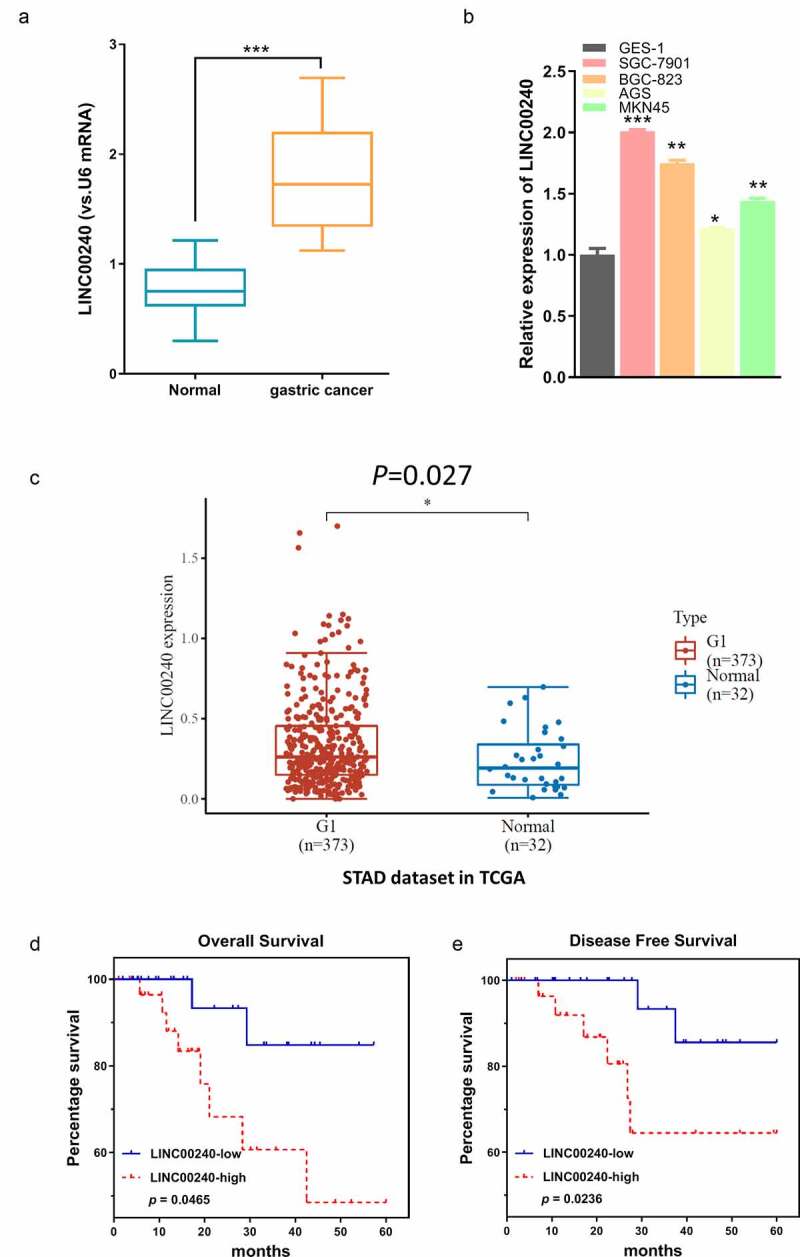
LINC00240 expression is increased both in GC tissues and GC cells. (a and b) qRT-PCR analysis showed the increased LINC00240 expression in GC tissues (n = 60). (a) and four GC cell lines (b) (*P < 0.05, **P < 0.01, ***P < 0.001). (c) Upregulation of LINC00240 expression in GC tissues from TCGA datasets. (d and e) High LINC00240 expression is associated with lower overall survival (d) and progression free survival (e) in GC patients

We first determined the expression level of LINC00240 between the GC tissues and adjacent normal tissues (n = 60). qRT-PCR analysis showed that LINC00240 was upregulated in GC tissues as compared to adjacent normal tissues (Figure 1(a)). Consistently, LINC00240 was also significantly upregulated in four GC cell lines (SGC-7901, BGC-823, AGS and MKN45) as compared to normal gastric epithelial cells (GES 1) (Figure 1(b)). We also analyzed the expression level of LINC00240 in both GC and normal tissues from TCGA database. LINC00240 was significantly upregulated in GC tissues as compared to the normal ones (Figure 1(c)). We further divided the patients into low expression (n = 30) and high expression group (n = 30) based on the median expression level of LINC00240. Kaplan-Meier survival analysis revealed that the overall survival (OS) and progression-free survival (PFS) were poorer in LINC00240 high expression group (Figure 1(d,e)). The association of LINC00240 expression level with the clinicopathological characteristics of GC patients, such as age, gender, TNM stage, distant metastasis, and lymph nodes metastasis, were shown in Table 1. LINC00240 high expression group was associated advanced TNM stage, a higher extent of distant metastasis and lymph nodes metastasis (Chi-square test, *p *< 0.05). However, no significant correlation was found between the level of LINC00240 and patient gender and age (Chi-square test, *p *> 0.05). Collectively, these data suggest that high LINC00240 expression favors GC progression.

**Table 1.** Correlations of LINC00240 expression with clinicopathological features of gastric cancer

		LINC00240 expression		
Variable	Number	Low (n = 30)	High (n = 30)	*P*-value
Age (years)				0.68
< 60	21	10	11	
≥ 60	39	20	19	
Gender				0.93
Male	32	18	14	
Female	28	12	16	
TNM				0.02
I–II	26	20	6	
III–IV	34	10	24	
Distant metastasis				0.02
No	33	22	11	
Yes	27	8	19	
Lymph nodes metastasis				0.03
No	32	21	11	
Yes	28	9	19

### 3.2. LINC00240 knockdown impairs the malignant phenotype of GC cells

**Figure 2.**
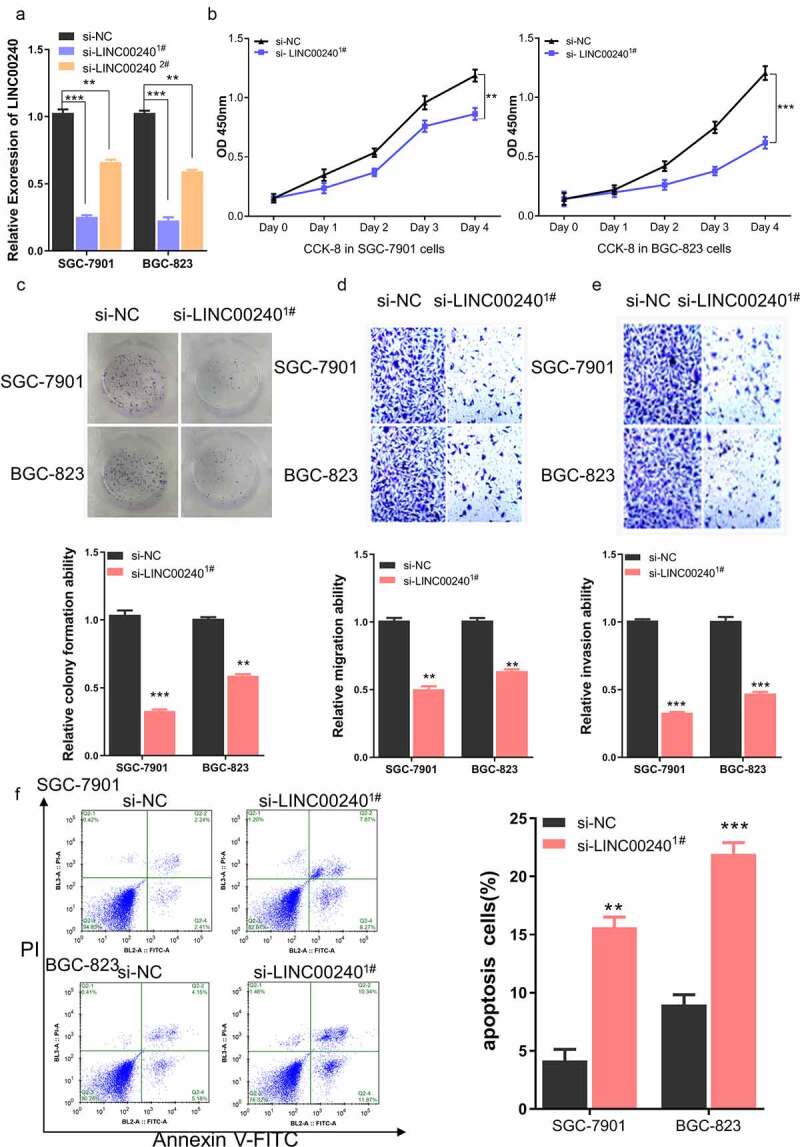
Knockdown of LINC00240 inhibits the proliferation and migration, and promotes cell apoptosis. (a) LINC00240 expression was quantified by qRT-PCR in GC cells (SGC-7901 and BGC-823) after siRNA transfection. (b) CCK-8 assay of the cell proliferation after knocking down LINC00240 in GC cells. (c) Colony formation assay, transwell migration (d) and invasion assay (e) in GC cell after siRNA transfection. (f) Flow cytometry analysis of apoptosis after LINC00240 knockdown (n = 3, **P < 0.01, ***P < 0.001)

To investigate the functional role of LINC00240 in the cellular phenotype of GC, we silenced LINC00240 in two GC cell lines (SGC-7901 and BGC-823) using siRNAs. Two siRNAs targeting LINC00240 (si-LINC00240^1#^ and si-LINC00240^2#^) and negative controls (si-NC) were transfected into GC cells. In both cell lines, the expression of LINC00240 decreased after the transfection of si-LINC00240^1#^ and si-LINC00240^2#^ as compared to the control (si-NC) group (Figure 2(a)). Since si-LINC00240^1#^ showed a stronger knockdown effect, si-LINC00240^1#^ was selected for the subsequent experiment. CCK-8 proliferation assay demonstrated that LINC00240 knockdown significantly impaired cell proliferation in both cell lines (Figure 2(b)). Moreover, the colony formation ability (Figure 2(c)), cell migration ability (Figure 2(d)) and cell invasion ability (Figure 2(e)) were significantly suppressed after LINC00240 knockdown. We further detected apoptosis level using flow cytometry and the results showed that knocking down LINC00240 increased the percentage of apoptotic cells (Figure 2(f)). Together, these data imply that LINC00240 is indispensable for the malignant phenotype of GC cells.

### 3.3. LINC00240 acts as a sponge for miR-338-5p

**Figure 3.**
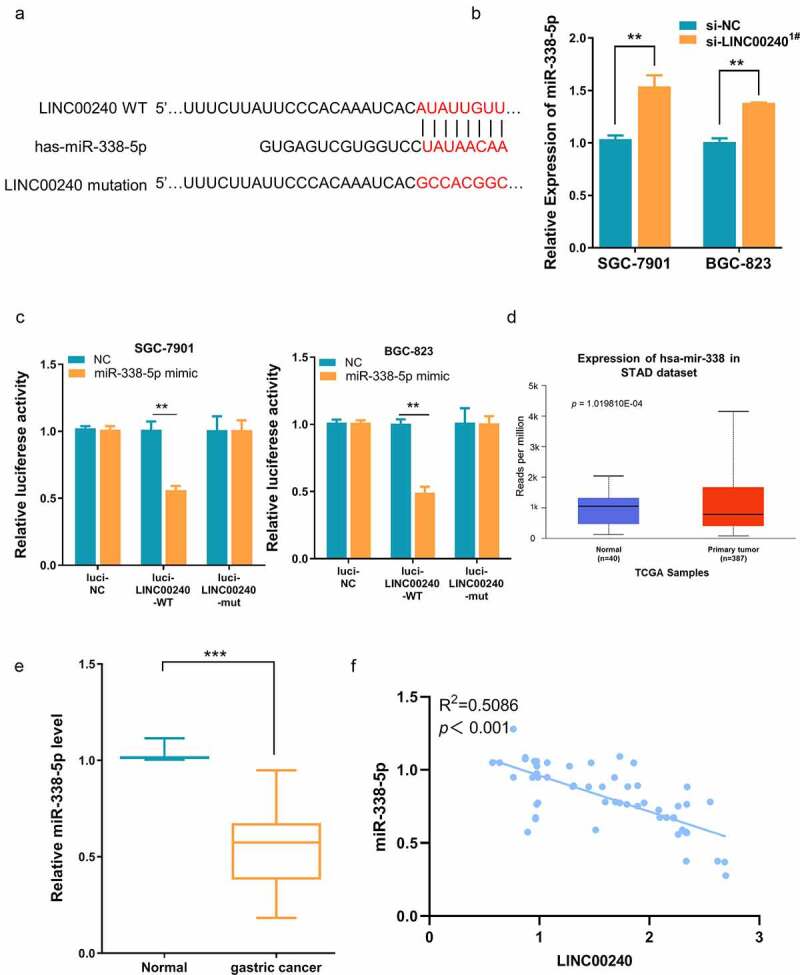
Evaluation the binding site between LINC00240 and miR-338-5p. (a) The binding site between LINC00240 and miR-338-5p based on bioinformatics analysis. (b) Increased expression level of miR-338-5p after LINC00240 knockdown detected by qRT-PCR. (c) Dual luciferase reporter assay using empty luciferase vector (luci-LINC00240-NC), wild type reporter with binding site (luci-LINC00240-WT), and the vector containing mutated binding sequence (luci-LINC00240-MUT) in the presence of miR-338-5p mimic. (n = 3, **P < 0.01). (d and e) Downregulation of miR-338-5p expression in GC tissue from TCGA datasets (d) and the GC tissues collected in this study (e). (f) Negative correlation between LINC00240 and miR-338-5p expression in GC tissues collected in this study (R^2^ = 0.5086, *p* < 0.001)

To further investigate the potential interaction between LINC00240 and miR-338-5p, a binding site between LINC00240 and miR-338-5p was predicted using DINAN online tools (http://carolina.imis.athena-innovation.gr/diana_tools/web/index.php?r=lncbasev2%2Findex). (Figure 3(a)). The knocking down of LINC00240 could significantly increase the expression of miR-338-5p (Figure 3(b)). Furthermore, we performed dual-luciferase reporter assay and found that, the luciferase activity of wild-type reporter (luci-LINC00240-WT) was significantly inhibited by miR-338-5p mimic. However, no significant change was observed in the empty luciferase vector (luci-LINC00240-NC) or the vector containing mutated binding sequence (luci-LINC00240-MUT) (Figure 3(c)). We further analyzed the expression level of miR-338-5p in both GC and normal tissues using data from TCGA database. miR-338-5p was significantly downregulated in GC tissues (Figure 3(d)). In the patient samples collected, we also found that miR-338-5p expression was significantly reduced in GC tissues as compared to the adjacent normal tissues (Figure 3(e)). The Spearman correlation analysis further showed that there was a significant negative correlation between the expression of miR-338-5p and LINC00240 (Figure 3(f)). Together, the above data indicate that LINC00240 acts as a sponge to negatively regulate miR-338-5p.

### 3.4. miR-338-5p mediates the inhibitory effect of LINC00240 on GC cells

**Figure 4.**
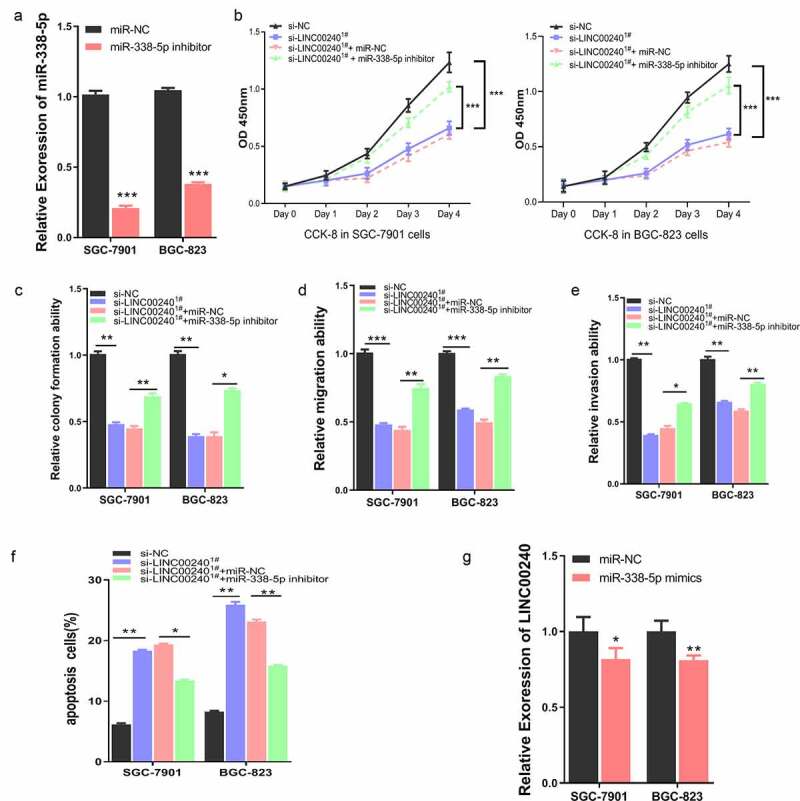
miR-338-5p inhibitor rescues si-LINC00240-induced inhibition on GC cell function. (a) qRT-PCR showed the downregulation of miR-338-5p by miR-338-5p inhibitor. (b-e) miR-338-5p inhibitor rescued si-LINC00240-induced inhibition on cell proliferation by CCK-8 assay (b), colony forming assay (c) and transwell migration and invasion assay (d and e). (f) miR-338-5p inhibitor reduced the level of apoptosis induced by si-LINC0024. (g) miR-338-5p mimic marginally downregulated LINC00240 level in GC cells. (n = 3, *P < 0.05, **P < 0.01, ***P < 0.001)

To further explore the interplay of LINC00240 and miR-338-5p on cell function, we applied miR-338-5p inhibitor, which could significantly reduce the level of miR-338-5p (Figure 4(a)). We next assessed whether miR-338-5p inhibitor could affect the function of LINC00240. We co-transfected the cells with LINC00240 siRNA, miR-338-5p inhibitor or miRNA-NC. Interestingly, the inhibitory effect of LINC00240 silencing could be rescued by miR-338-5p inhibitor, but not by miRNA-NC (Figure 4(b)). Furthermore, miR-338-5p inhibitor could also alleviate the si-LINC00240-induced inhibition on colony formation, cell migration and invasion (Figure 4(c–e)). In the meanwhile, miR-338-5p inhibitor attenuated the cell apoptosis induced by LINC00240 silencing (Figure 4(f)). Interestingly, we found that the application of miR-338-5p mimic only marginally reduce the expression level of LINC00240 (Figure 4(g)), suggesting that the effect of miR-338-5p on LINC00240 silencing may not mainly due to its effect on LINC00240 expression.

### 3.5. METTL3 is negatively regulated by miR-338-5p

**Figure 5.**
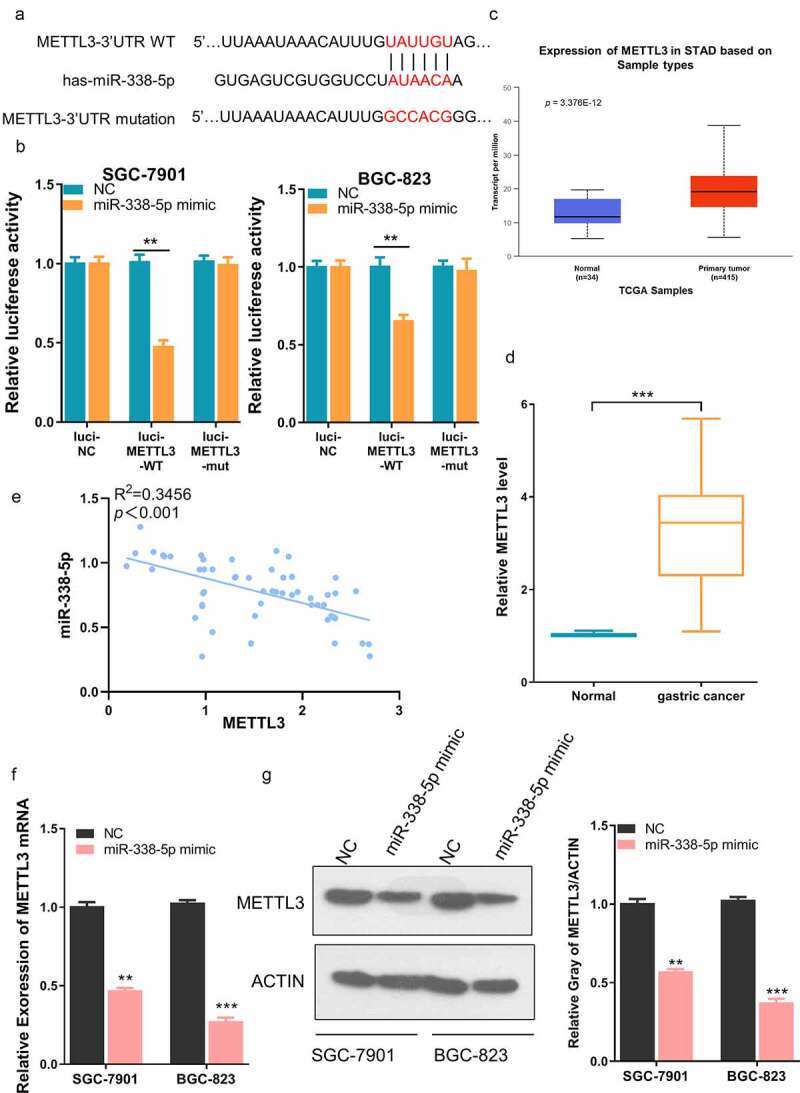
miR-338-5p targets METTL3 expression in GC cells. (a) The binding site between miR-338-5p and METTL3 3ʹ UTR from bioinformatics analysis. (b) Dual luciferase reporter assay using empty luciferase vector (luci-METTL3-NC), wild type reporter with binding site (luci-METTL3-WT), and the vector containing mutated binding sequence (luci-METTL3-MUT) in the presence of miR-338-5p mimic. (n = 3, ***P < 0.001). (c) Upregulation of METTL3 expression in GC tissue from TCGA datasets and (d) GC tissues collected in this study. (e) Negative correlation between METTL3 and miR-338-5p expression level in GC tissues collected in this study (R^2^ = 0.3456, *p* < 0.001). (f and g) Downregulation of METTL3 by miR-338-5p mimic as measured by qRT-PCR (f) and Western Blot assay (g) in SGC-7901 and BGC-823 cells (n = 3, **P < 0.01, ***P < 0.001)

To further search for the downstream target of miR-338-5p, we searched TargetScan database (http://www.targetscan.org/vert_72/), and found a potential binding site between miR-338-5p and METTL3 3ʹ UTR (untranslated region) (Figure 5(a)). We performed dual-luciferase reporter assay using reporter containing wild-type binding sequence (luci-METTL3-WT), empty luciferase vector (luci-METTL3-NC) or the vector containing mutated binding sequence (luci-METTL3-MUT). The presence of miR-338-5p mimic significantly inhibited the activity of wild-type reporter while had no effect on empty vector or reporter containing mutated bindings sequence (Figure 5(b)). According to the TCGA database analysis, METTL3 was significantly upregulated in GC tissues (Figure 5(c)). We also showed that METTL3 displayed a significantly higher expression level in our GC tumor samples, and there was a negative correlation between the expression of METTL3 and miR-338-5p (Figure 5(d,e)). Furthermore, we showed that miR-338-5p mimic significantly reduced METTL3 at mRNA and protein level GC cell lines (Figure 5(f,g)). Collectively, the above data demonstrated that METTL3 is negatively regulated by miR-338-5p.

### 3.6. LINC00240 controls malignant phenotype of GC cells by regulating miR-338-5p/METTL3 axis

**Figure 6.**
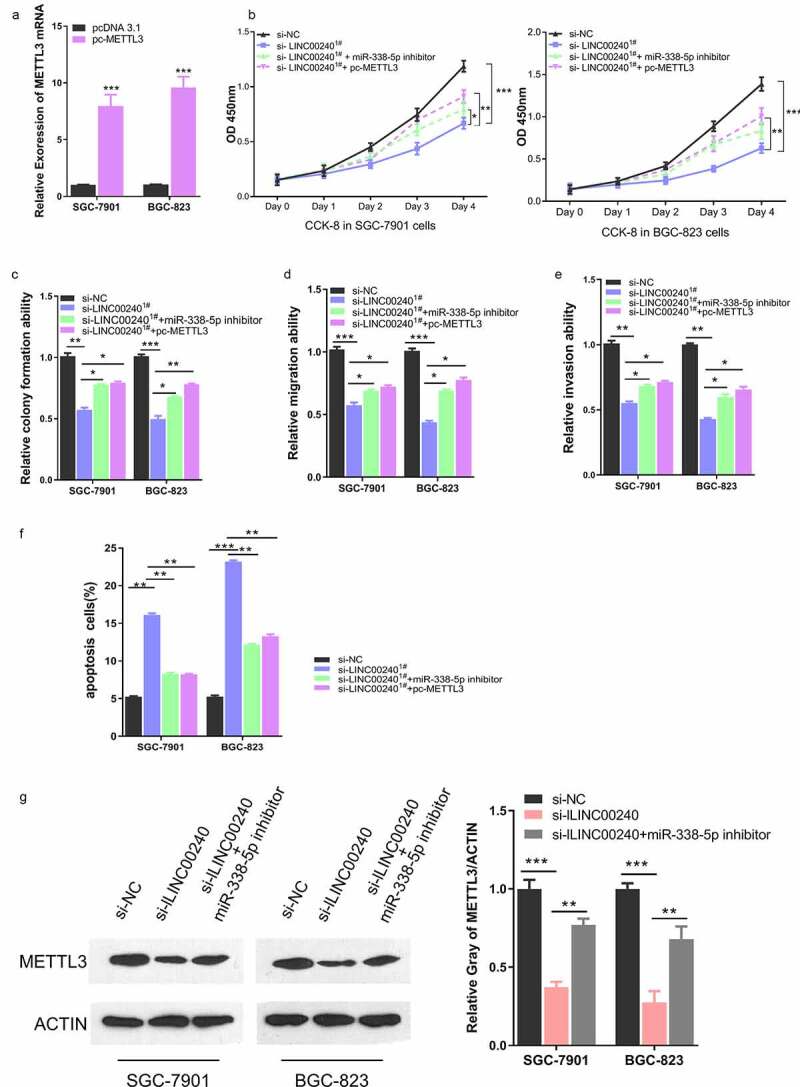
METTL3 overexpression rescues si-LINC00240-induced inhibition on GC cell function. (a) Overexpression of METTL3 after the transfection of pc-METTL3 expression vector. (b-f) GC cells were transfected with si-NC, si-lLINC00240, si-lLINC00240+ miR-338-5p inhibitor, or si-lLINC00240+ pc-METTL3. (b) CCK-8 Cell proliferation, (c) colony formation, (d) cell migration, (e) cell invasion, and (f) apoptosis assays were performed in GC cells 48 hours after the transfection. (g) Expression of METTL3 was measured by Western Blot after transfecting with si-lLINC00240 or si-lLINC00240+ miR-338-5p inhibitor. (n = 3, *P < 0.05, **P < 0.01, ***P < 0.001)

To further verify that the functional role of LINC00240 malignant phenotype of GC cells depends on miR-338-5p/METTL3 axis, we further constructed METTL3 expression vector (pc-METTL3) by cloning the cDNA of METTL3 into pcDNA 3.1 vector. Transfection of pc-METTL3 could significantly upregulate METTL3 in both cell lines (Figure 6(a)). We then assessed whether METTL3 overexpression recapitulated the rescue effect of miR-338-5p inhibitor on LINC00240 silencing. As expected, silencing of LINC00240 suppressed cell proliferation, which was rescued by miR-338-5p inhibitor (Figure 6(b)). METTL3 overexpression also recued the inhibitory effect of LINC00240 silencing on cell proliferation. Furthermore, METTL3 overexpression could also alleviate the si-LINC00240-induced inhibition on colony formation, cell migration and invasion (Figure 6(c–e)), and attenuated the cell apoptosis induced by LINC00240 silencing (Figure 6(f)). Furthermore, we investigated whether LINC00240/miR-338-5p axis controls the expression of METTL3. Western Blot analysis showed that METTL3 level was downregulated after LINC00240 knockdown, which could be partially restored by miR-338-5p inhibitor (Figure 6(g)). Collectively, our data indicates that miR-338-5p/METTL3 axis play an essential role in mediating the functional role of LINC00240 in GC cells.

## 4. Discussion

Previous studies suggest that lncRNAs can either function as oncogenes or tumor suppressor genes [23]. Accumulating evidence highlighted the essential roles of lncRNAs in regulating the pathophysiological processes of GC. For example, Luo et al. found that lncRNA MYLK antisense RNA 1 (MYLK-AS1) is overexpressed in GC cell, which negatively regulates the expression of large tumor suppressor 2 (LATS2) and promotes cell proliferation and invasion by binding with enhancer of zeste homolog 2 (EZH2) [24]. Hu et al. found that the overexpression of lncRNA LINC01320 contributes to the aggressive behaviors of GC cells via modulating miR-495-5p/RAB19 axis [25]. Moreover, they showed that the upregulation of LINC01320 is induced by METTL14-mediated m6A modification.

In our study, we demonstrated the upregulation of lncRNA LINC00240 in GC tissues and GC cell lines, and its essential role in supporting the malignant phenotype of GC cells. High expression of LINC00240 was associated with advanced TNM stage, a higher extent of distant metastasis and lymph nodes metastasis, and the poor overall and disease-free survival of the patients. These data suggest that LINC00240 upregulation may favor the malignant progression of GC. The increased expression of LINC00240 has been reported in different cancers, including non-small cell lung cancer [26], hepatocellular carcinoma cells [27], cervical cancer [28], and gastric cancer cell [10]. Consistent with our results, Li et al. showed that LINC00240 was upregulated in GC tissues, which is correlated with poor prognosis of GC patients [10].

We investigated the functional interactions between LINC00240 and the downstream targets. We demonstrated that LINC00240 could serve as a sponge to negatively regulate the activity of miR-338-5p. Besides, we found a negative correlation between the expression of LINC00240 and miR-338-5p in GC tissues, and a negative correlation between the expression of miR-338-5p and METTL3. Consistently, previous Studies also reported that the expression level of miR-338-5p and miR-338-3p were reduced in GC tissues and cells [14,16,29]. In contrast, METTL3 overexpression has been proposed as a promoter for GC metastasis and progression in multiple studies [18,21,30]. High METTL3 level seems to facilitate GC progression and metastasis by promoting epithelial–mesenchymal transition (EMT) and tumor angiogenesis [18–21]. In line with this notion, our study showed that the expression level of miR-338-5p was increased in GC cells after LINC00240 knockdown, while METTL3 level was reduced after LINC00240 silencing. Importantly, miR-338-5p inhibitor or METTL3 overexpression could rescue the inhibitory effect of LINC00240 knockdown on cell proliferation and migration, and inhibit the apoptosis induction in GC cells. Taken together, our data suggest that the upregulation of LINC00240 in GC cells promotes the malignant phenotype by impairing the activity of miR-338-5p and maintaining a high level of METTL3.

Exploring novel regulatory mechanisms is critical for understanding the complex biology behind GC progression. The expanding roles of lncRNA-mediated axis in controlling GC phenotype begin to emerge. Li et al. identified LINC00240/miR-124-3p/DNMT3B as a novel regulatory module in GC progression [10]. Tong et al. demonstrated that GC cell proliferation is modulated via MECP2/miR-338/P-REX2 or BMI1 pathway [14]. Our results revealed that LINC00240 plays a critical role in promoting GC cell proliferation and invasion via miR-338-5p/METTL3 axis, which enriches the regulatory network of LINC00240 and provides potential therapeutic targets for GC treatment.

## 5. Conclusion

LINC00240/miR-338-5p/METTL3 axis has been delineated in this study as a functional module regulating the malignancy of GC cells. We found that LINC00240 is upregulated in both GC tissues and cells, and its upregulation is associated with poor survival of GC patients. We further demonstrated that increased LINC00240 expression suppresses miR-338-5p expression and maintain a high level of METTL3 in GC cells, which is essential to support the malignant phenotype of GC cells. Future animal model study is required to validate LINC00240/miR-338-5p/METTL3 axis in the tumorigenesis and progression of GC.

## Highlights

- Long non-coding RNA LINC00240 promotes the progression of gastric cancer.
- High expression of LINC00240 is associated with poor survival of gastric cancer patients.
- LINC00240 promotes gastric cancer by regulating miR-338-5p/METTL3 axis.
